# The Relationship of Cytokines IL-13 and IL-17 with Autoantibodies Profile in Early Rheumatoid Arthritis

**DOI:** 10.1155/2016/3109135

**Published:** 2016-08-04

**Authors:** Isabela Siloşi, Mihail Virgil Boldeanu, Manole Cojocaru, Viorel Biciuşcă, Vlad Pădureanu, Maria Bogdan, Ramona Georgiana Badea, Carmen Avramescu, Ileana Octavia Petrescu, Florin Petrescu, Cristian A. Siloşi

**Affiliations:** ^1^Department of Immunology, University of Medicine and Pharmacy of Craiova, 2 Petru Rares Street, 200349 Craiova, Romania; ^2^Department of Physiology, Titu Maiorescu University of Bucharest, 187 Calea Vacaresti Street, 004051 Bucharest, Romania; ^3^Department of Medical Semiology, University of Medicine and Pharmacy of Craiova, 2 Petru Rares Street, 200349 Craiova, Romania; ^4^Department of Pharmacology, University of Medicine and Pharmacy of Craiova, 2 Petru Rares Street, 200349 Craiova, Romania; ^5^Medico Science SRL, Stem Cell Bank Unit, 1B Brazda lui Novac Street, 200690 Craiova, Romania; ^6^Department of Microbiology, University of Medicine and Pharmacy of Craiova, 2 Petru Rares Street, 200349 Craiova, Romania; ^7^Department of Pediatrics, University of Medicine and Pharmacy of Craiova, 2 Petru Rares Street, 200349 Craiova, Romania; ^8^Department of Surgery, University of Medicine and Pharmacy of Craiova, 2 Petru Rares Street, 200349 Craiova, Romania

## Abstract

*Aims. *In the present study, we aimed to assess the concentrations of IL-13 and IL-17 in serum of patients with early rheumatoid arthritis (eRA), the investigation of correlation between the concentrations of these cytokines and disease activity score, and the concentration of some autoantibodies and the evaluation of the utility of IL-13 and -17 concentration measurements as markers of disease activity.* Materials and Methods*. Serum samples were collected from 30 patients and from 28 controls and analysed parameters.* Results*. The serum concentrations of IL-13, IL-17, anti-CCP, and IgM-RF were statistically significantly higher in patients with eRA, compared to the controls. IL-13 concentrations in the severe and moderate groups with eRA were statistically higher than in the mild and control groups. Also, in the case of IL-17, serum concentrations increased proportionally with the disease activity of eRA. We observe that concentrations of IL-13 and -17 did not correlate with autoantibodies. IL-17 concentration significantly positively correlated with CRP, while IL-13 concentration significantly negatively correlated with CRP. Disease activity score, DAS28, was strongly positively correlated with levels of ESR and weakly positively correlated with concentrations of anti-RA33 autoantibodies. IL-13 has a higher diagnostic utility than IL-17, CRP, ESR, IgM-RF, and anti-CCP as markers of disease activity.* Conclusions*. The presence of higher IL-13 and IL-17 serum levels in patients, compared with those of controls, confirms that these markers, found with high specificity, might be involved in the pathogenesis of eRA. IL-13 and IL-17 might be of better usefulness in the prediction of eRA activity status than IgM-RF and anti-CCP.

## 1. Background and Aims 

Rheumatoid arthritis (RA) is a chronic, progressive, inflammatory autoimmune disease in which the body's immune system mistakenly attacks the joint. The disease produces an inflammatory infiltrate of immune cells as well as a series of destructive events such as synovial hyperplasia, pannus setting, bone and cartilage erosion, and joint destruction. It results in swelling and pain in the joints and around them [1, 2].

In RA, activation of innate immunity in early disease, followed by the appearance of adaptive immune responses ultimately leads to a destructive phase. The pathophysiology of RA implies the existence of T and B cells, various immune modulators (cytokines and effector cells), and signalling pathways. The complex interaction of immune modulators causes joint damage starting at the synovial membrane and covering most structures [3].

This disequilibrium between pro- and anti-inflammatory cytokine activities facilitates the induction of autoimmunity, chronic inflammation, and joint damage. It is less known, though, how cytokines are organized within a hierarchical regulatory network and which cytokines may qualify as best targets for clinical intervention a priori [3–5].

RA pathogenesis is caused by B cells not only through antigen presentation, but also through the production of antibodies, autoantibodies, and cytokines [2, 5].

There are no disease-specific diagnostic features in RA and patients can have a wide range of manifestations. The diagnosis of RA is given by a combination of symptoms, signs, serologic tests, and radiologic findings, as established by the American College of Rheumatology [6].

Since early inflammatory arthritis is a clinically heterogeneous disease, cytokine networks are known to play a critical role in the pathogenesis of rheumatoid arthritis; a panel of pro- and anti-inflammatory cytokines and associated autoantibodies were measured to identify the biologically based subsets of early rheumatoid arthritis (eRA) [7].

Emery and Symmons [8] discuss in their article the difficulties of early diagnosis. They said that diagnosis of early rheumatoid arthritis (RA) has inherent difficulties. It requires assessment, not only of the current clinical picture but also of the potential for change.

Therefore, identifying the early rheumatoid arthritis (RA) is a crucial step in controlling the progress of the disease. A major outcome will be achieved through early diagnosis and treatment [9].

Early diagnosis of rheumatoid arthritis (RA) is essential, because there is strong evidence that early treatment with one or more disease-modifying antirheumatic drugs improves the evolution [10, 11]. When DMARDs therapy is introduced early in patients, function and radiological outcome in the long term are better than cases when it is delayed [12].

Our research objectives were to assess the concentrations of IL-13 and IL-17 in serum of patients with eRA, the investigation of correlation between the concentrations of these cytokines and disease activity score and the concentration of some autoantibodies in relation to the control group, and the evaluation of the utility of IL-13 and IL-17 concentration measurements as markers of disease activity.

## 2. Materials and Methods

### 2.1. Subjects and Clinical Assessment

We accomplished a study which included 30 patients diagnosed with early rheumatoid arthritis, gender ratio 6 M/24 F, and mean age 56.22 years; in parallel, we investigated a control group that included 28 persons unaffected by early rheumatoid arthritis or other diseases. Controls were matched for sex, age at the time point of blood sampling, and area of residence (rural or urban).

Early RA patients fulfilled the American College of Rheumatology (ACR) 1987 revised criteria for the classification of RA [13]. They were all investigated, diagnosed, and included in the studied group, following the revised classification criteria of the American College of Rheumatology, in 2010 [6]. All patients accomplished the inclusion criteria for early rheumatoid arthritis (two or more swollen joints dating more than 2 weeks, but less than 12 months from onset).

We excluded, from the start of the study, patients with other autoimmune diseases, those who received treatment with DMARDs, glucocorticoids, or/and vitamins, the women during pregnancy, and persons with diabetes mellitus or metabolic syndrome. The study cohort comprised patients firstly evaluated for early arthritis because we aimed to investigate eRA patients to find a better and faster way of discrimination between affected and unaffected cases.

Based on the DAS28 results, the 30 eRA patients were subdivided into three groups: mild (2.6 < DAS28 ≤ 3.2), moderate (3.2 < DAS28 ≤ 5.1), and severe (5.1 < DAS28).

Serum samples were collected from 30 patients and from 28 controls (healthy persons) and analyzed for concentrations of IL-13 and IL-17, anticyclic citrullinated peptide antibodies (anti-CCP), rheumatoid factor IgM isotype (IgM-RF), anti-cardiolipin IgG isotype (IgG-aCL), anti-RA33, erythrocytes sedimentation rate (ESR), and C-reactive protein (CRP).

### 2.2. Samples Collection

Blood samples were obtained from all subjects into tubes without additives by venous puncture in a fasting state in the morning. Peripheral venous blood was collected into separator vacutainers and allowed to clot for 30 minutes at room temperature. The test tubes were centrifuged at 3.000 ×g for 10 minutes, and serum samples were further divided into aliquots and stored at −80°C, until assessment. Before testing, frozen probes were brought to room temperature, avoiding freezing-unfreezing cycles.

### 2.3. Immunological Investigations

The analysis of serum parameters was based on a quantitative sandwich ELISA, according to the manufacturer's instructions. IgG anti-CCP 3.1 and IgG-aCL autoantibodies were determined by ELISA, using Quanta Lite*™*-INOVA Diagnostics kits, USA (autoantibodies seropositivity was defined as a titer >20 U/L). The investigation of serum IgM-RF concentrations was achieved using ELISA-AESCU Germany kits (positive >15 U/L) and of anti-RA33 antibodies using ELISA-kit Human, Wiesbaden, Germany (positive results >25 U/mL). For hsCRP dosage, DRG ELISA International Inc. USA kit was used (the positive values were >10 mg/L).

Serum concentrations of IL-13 and IL-17 were measured in patients with early, untreated inflammatory arthritis and in control persons, using ELISA techniques with Invitrogen Corporation kits (Camarillo, CA, USA). The values obtained were expressed in pg/mL. In looking for a method of measuring serum cytokine, concentrations were taken into account studies that show good stability for samples stored at −70°C until dosage [14]. Most manufacturers recommended avoiding repeated freeze-thaw cycles for serum samples.

All the procedures were followed in accordance with the ethical standards of the institutional responsible committees for human studies and with the Helsinki Declaration of 1975, as revised in 2008.

For realisation of this study, we obtained the approval of the Committee of Ethics and Academic and Scientific Deontology of the University of Medicine and Pharmacy from Craiova number 76/2014.

### 2.4. Statistical Analysis

Patients' data, management system, and data processing were performed using Microsoft Excel and Data Analysis module; statistical analysis was done using* GraphPad Prism 5*. All tests were two-sided and *p* values ≤ 0.05 were considered significant.

The significance of differences between groups was examined with a Mann-Whitney *U* test or Kruskal-Wallis, when multiple comparisons were made. Correlation analysis between the concentration of IL-13 and IL-17 and the degree of disease activity (DAS28), as well as the concentration of some autoantibodies, CRP and ESR, were conducted with a Pearson's test. All tests were two-sided and *p* values ≤ 0.05 were considered significant.

The diagnostic values of studied markers were evaluated using receiver operating characteristic (ROC) curves analysis. The performance was expressed as the area under the ROC curve (AUC, area under ROC curve) together with 95% confidence interval (95% CI) and *p* statistics for the difference between calculated AUC and AUC = 0.5 (weak discriminative marker). Cut-off values corresponding to the highest accuracy were determined and for various threshold values investigated at each marker, we calculated the sensitivity (Sn), specificity (Sp), and Youden index (sensitivity + specificity − 1).

## 3. Results

### 3.1. Clinical Characteristics of the Study Subjects

Among the 30 patients, initially diagnosed with eRA, 80% were female (sex ratio: 24 female/6 male), with age, mean ± stdev 55.77 ± 10.87 years. In controls group, incidence for women was 78% and age was 52.36 ± 13.38. There was no significant difference in age between the two groups (Table 1).

### 3.2. Cytokines Concentrations and Disease Activity Stage

In our study, we found that both IL-13 (18.20 pg/mL, 95% CI: 16.47–19.92) and IL-17 (17.87 pg/mL, 95% CI: 12.99–22.75) concentrations in the serum of patients suffering from eRA were higher than those in the control group (4.80 pg/mL, 95% CI: 3.89–5.71, *p* < 0.0001, and 4.20 pg/mL, 95% CI: 3.36–5.05, *p* < 0.0001, resp.).

We also found differences in serum concentrations of IL-13 and IL-17 in subgroups of eRA disease patients with different clinical activity stages.

**Figure 1:**
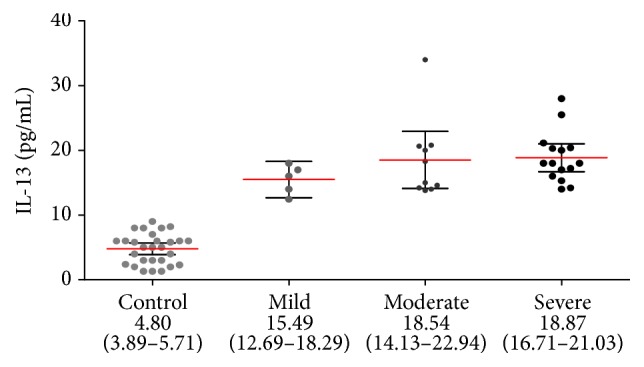
IL-13 concentration in serum of eRA disease patients with different clinical activity stages (black circles represent IL-13 concentration, pg/mL in individual serum samples; red lines represent mean values accompanied by 95% confidence interval, represented as black horizontal bars).

IL-13 concentrations were increasing along with the disease activity (Figure 1). The concentrations of IL-13 in the severe and moderate groups were statistically higher than in the mild and control groups (*p* < 0.05). There were no statistical differences between severe and moderate groups.

**Figure 2:**
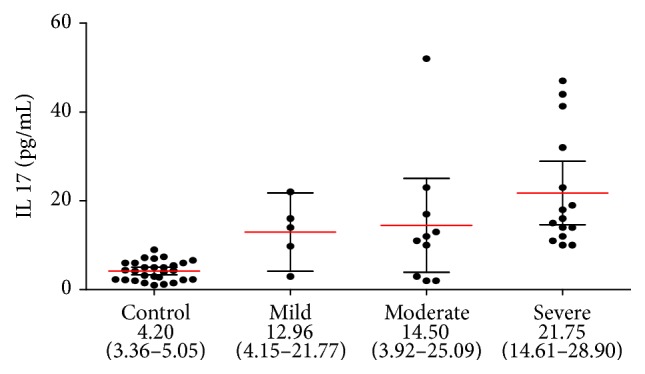
IL-17 concentration in serum of eRA disease patients with different clinical activity stages (black circles represent IL-17 concentration, pg/mL in individual serum samples; red lines represent mean values accompanied by 95% confidence interval, represented as black horizontal bars).

Also, in the case of IL-17 serum concentrations increased proportionally with the disease activity of eRA, the highest concentrations were in patients with severe activity disease (Figure 2). Statistically significant differences were observed between both the moderate and the severe groups and a mild group (*p* < 0.05), as well as between the group with moderate disease activity and the control group (*p* < 0.001).

In the studied cohort of patients, we observe statistically significant differences in the concentrations of CRP and the levels of ESR between patients with eRA and the control group (CRP/control group, *p* < 0.0001; ESR/control group, *p* < 0.0001) (Table 1). Analyzing the relationship between serum levels of CRP and ESR and different disease activity stages, we observed only statistically significant differences between severe and moderate group (*p* < 0.0379) (Table 2).

### 3.3. Autoantibodies Concentrations and Disease Activity Stage

Another objective of this study was to investigate autoantibodies profile in eRA. We reproduced in Table 2 concentrations of these autoantibodies investigated.

Following the analysis, our study showed different profiles of IgG anti-CCP and IgM-RF concentrations in serum of patients suffering from eRA in different clinical activity stages. IgG anti-CCP and IgM-RF concentrations were increasing along with the disease activity. In both cases, there were statistically significant differences between severe groups and moderate and mild groups (IgG anti-CCP: severe versus moderate group, *p* = 0.0011, severe versus mild group, *p* = 0.0030; IgM-RF: severe versus moderate group, *p* = 0.0014, severe versus mild group, *p* = 0.0039).

### 3.4. Correlations between IL-13, IL-17, and Indices of eRA

Concentrations of both interleukins are not correlated with each other (Table 3). Also we observe that concentrations of IL-13 and IL-17 are not correlated with autoantibodies. There was a weak, statistically not significant correlation between IL-17 and IgM-RF (*r* = 0.320, *p* = 0.085).

There was a significant positive correlation between the concentrations of IL-17 and CRP (*r* = 0.366, *p* = 0.047) and a significant negative correlation between the concentrations of IL-13 and CRP (*r* = −0.334, *p* = 0.041).

Disease activity score, DAS28, was strongly positively correlated with levels of ESR (*r* = 0.967, *p* ≤ 0.001) and weakly positively correlated with concentrations of anti-RA33 autoantibodies (*r* = 0.404, *p* = 0.027).

Concentrations of anti-CCP autoantibodies positively correlated fairly well with CRP and IgM-RF, and concentrations of anti-RA33 positive correlated with levels of ESR.

### 3.5. Diagnostic Performance of IL-13 and IL-17 as Disease Markers

Comparing the ROC curves for the studied parameters in the patients with eRA indicated that IL-13 has a higher diagnostic utility than IL-17, CRP, ESR, IgM-RF, and anti-CCP as markers of disease activity (Table 4).

**Figure 3:**
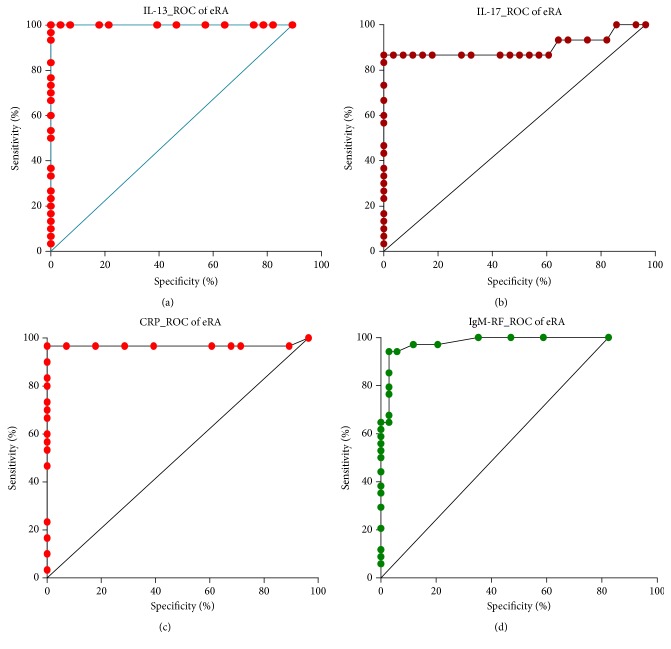
Comparison of ROC curves for IL-13 (a), IL-17 (b), CRP (c), and IgM-RF (d).

ROC analysis revealed that IL-13 concentration indicated eRA presence with 100% accuracy using the concentration of 10.73 pg/mL as an optimal cut-off value for discrimination between patients with eRA and controls (95% CI: 0.962–1.000, *p* < 0.0001). The likelihood ratios of positive and negative results obtained on the basis of optimal threshold values specific for eRA were as follows: LR(+) = 28.00 and LR(−) = 1.12 with sensitivity and specificity equal to 100 and 100%, respectively; Youden index was 1.00 (Figure 3).

In case of IL-17, the calculated cut-off value for discrimination between patients with eRA and controls was 9.40 pg/mL and using this value the diagnostic accuracy of IL-17 was 90.2% (95% CI: 0.809–0.995, *p* < 0.0001). The likelihood ratios of positive and negative results obtained on the basis of optimal threshold values specific for eRA were as follows: LR(+) = 24.27 and LR(−) = 1.14 with sensitivity and specificity equal to 86.67 and 100%, respectively; Youden index was 0.866.

In the studied cohort of patients, CRP and ESR have discriminative power towards diagnosis of eRA (sensitivities for both CRP and ESR were found to be lower in comparison to the IL-13; diagnostic accuracy of CRP was 96.9 and 94.8%, resp., for ESR).

We also noticed that although they have a specificity less than IL-13 and CRP, autoantibodies IgM-RF and anti-CCP have a good tendency to discriminate patients with eRA from healthy ones (diagnostic accuracy 98.1 and 94.7%, resp.).

## 4. Discussions

RA is an inflammatory autoimmune disease characterized by systemic and articular effects. Chronic inflammatory and autoimmune diseases are the result of an interplay between genetic factors and environmental ones that culminate in the phenotypes of the established disease. Owing to the prevalence and accessibility of joint samples for laboratory investigation, RA has been a suitable model for the study of numerous inflammatory and immune-mediated conditions [15].

The formulation of a definition for early RA was difficult, but the majority of the rheumatologists use the term “early” for symptoms shorter than three months. There was a tendency to accept the involvement of fewer affected joints [9, 16].

Changing from health to established disease in RA is generally clearly understood.Early rheumatoid arthritis (RA) and very early RA are major targets of research and clinical practice [15, 17].

We found a predominance (80%) of women in patients affected by eRA, finding which are congruent with the results of other studies having identified a female predominance in RA [9, 15, 18].

Both pro- and anti-inflammatory cytokines were found elevated in RA patients over controls claiming opinion that cytokine networks play critical rolls in the pathogenesis of rheumatoid arthritis [4, 7, 19–28].

The present study reveals that levels of serum IL-13 and IL-17 cytokines were significantly higher in eRA patients than in age- and sex-matched healthy persons. Our results are related to the cell-mediated immune response intervention in disease onset.

A broad range of inflammatory processes that are involved in the pathophysiology of rheumatoid arthritis are regulated by cytokines. The imbalance between pro- and anti-inflammatory cytokine activities favours the induction of autoimmunity, chronic inflammation, and thereby joint damage [5, 27].

Synovial inflammation is regulated by cytokines. Some cytokines, such as tumour necrosis factor- (TNF-) alpha, IL-17, and (IL)-1, function by promoting inflammatory responses and by inducing cartilage degradation. Others such as IL-4, IL-10, and IL-13 are mainly anti-inflammatory molecules [18, 20, 25].

Even if present in rheumatoid joints, in progressive RA anti-inflammatory cytokine levels are too low to neutralize the deleterious effects of proinflammatory cytokines. The suppression of both the secretion and action of IL-17 by IL-13 is of potential clinical importance [19–22].

IL-13 is a protein, secreted by activated T cells, that modulates B cell function in vitro and plays an important part in their proliferation and differentiation; the high local IL-13 levels were observed in patients with RA, correlated with B lymphocyte proliferation [23]. Interleukin 13 induces interleukin-4-independent IgG4 and IgE synthesis and CD23 expression by human B cells [24].

Some researchers support a role for IL-13 as an in vivo antiangiogenic factor and provide a rationale for its use in RA to control pathologic neovascularization [25].

Treatment with Th2 cytokines (IL-4, IL-10, and IL-13) was tested in many animal models of arthritis based on the Th1 bias of T cells, showing considerable promise [26].

In patients suffering from RA, disruptions in self-tolerance lead to abnormalities such as recognition of citrullinated antigens by T and B cells. The proportion of lymphocyte differentiation in RA is skewed towards the Th1 phenotype, to the detriment of the Th2, Th17, and T regulator (Treg) ones. Imbalances appear in the main cytokine systems including IL-1, TNF, IL-6, IL-18, IL-15, IL-33, IL-22, and IL-13. However, the destruction of the joint in RA is caused not only by these cytokine imbalances but also by matrix production dysregulation responsible for cartilage damage.

IL-17 levels fall after administration of anti-inflammatory cytokines such as IL-4 or IL-13 [27].

IL-13 levels were significantly higher in patients with early RA (*p* < 0.001) than in reference group, suggesting the different pathogenic mechanisms involved in joint inflammation. Serum IL-13 values increased in RA have been reported in many works [20, 22, 28].

Lower interleukin 13 levels were communicated in patients with arthritis by some investigators as Barra and contributors [29] and Woods et al. [30].

Spadaro et al. [28] assume that the production of RF and antinuclear antibodies by B cells could depend on different cytokines action. In their study, IL-13 serum levels correlated with those of RF in RA patients and they suggested that IL-13 may be involved in the pathogenesis of autoimmune rheumatic diseases, with a relevant role on RF production. McKenzie et al. [31] emphasize the involvement of IL-13 in regulating human monocyte and B cell function.

We found that detection of IL-13 in eRA patients was not affected by rheumatoid factor IgM (*r* = 0.206, *p* = 0.274), a fact revealed by other researchers too [32].

Interleukin 13 inhibits the production of proinflammatory cytokines, chemokines, and hematopoietic growth factors by activated human monocytes [30]. The increase of biologically active IL-13 in RA supports the hypothesis that IL-13 regulates immune cell (including dendritic cell) activity and indicates how the varied anatomical distribution of cytokines may play a role in the RA disease process. The differential regulation of circulating IL-13 and M-CSF levels by TNF antagonists further implies discrete roles in the TNF-cytokine network in RA [33, 34].

Isomäki et al. [34] showed that IL-13 was present in 27 out of 28 serum samples from patients with RA, indicating that this cytokine is constantly present in rheumatoid joints.

Raza et al. [35] detected increased levels of the Th2 cytokines IL-4 and IL-13 in synovial samples from early RA patients.

IL-13 causes B cell proliferation and differentiation, including IgE production, and the expression of certain adhesion molecules on endothelial cells. All these biological properties of IL-13 are shared with IL-4, but in contrast to IL-4, IL-13 does not act on T cells [36].

Besides the increased values of IL-13, we detected circulating IL-17 levels significantly higher in patients with eRA (*p* < 0.001) when compared to those in the reference group.

IL-17 concentrations were associated in ten patients with seropositivity for IgM-RF. Strong correlations of serum IL-17A levels with anti-CCP were found by Roşu et al. [18].

Th17 with decreased circulating levels in eRA seems to be a marker of anti-CCP seropositivity [37]. Considering the complexity and heterogeneous nature of RA, it is unlikely that only cytokines investigation may provide sufficient discrimination; predicting the eRA is better with a combination of biomarkers [38].

IL-17 has several sources: Th17 cells, which are a subset of CD4+helper T cells, mast cells, NK cells, and *γδ* T cells; all of them contributing to the pathogenesis of inflammatory arthritis [39, 40].

In a recent study comparing individuals before the onset of symptoms (defined as prepatients) and after the onset of RA with matched control subjects, Kokkonen et al. [41] showed that IL-17 was present at its highest concentrations in prepatients, and the level had decreased within 7.7 months following the onset of disease.

IL-17A was detected at higher levels in early disease compared with late, established disease [42]. Roşu et al. [18] reported IL-17A levels significantly higher in synovial fluid (SF) and serum from eRA patients compared to osteoarthritis (OA). In other previous studies, there were higher serum and SF IL-17A levels in RA patients as compared to healthy controls, which suggests that the cytokine is mainly produced locally in the inflamed joint [43].

Other results sustain that despite the significant increases in Th17 and IL-17 CD4+ T cells in the blood of RA patients, these did not correlate with ESR, CRP, or DAS28, suspecting that the presence of IL-17 producing CD4+ T cells in the blood from patients with established RA is of limited use as a biomarker to indicate disease activity [44]. In our study, there were significant correlations (*r* = 0.366, *p* = 0.047) found between IL-17 and CRP serum levels, but not with ESR or disease activity score, supporting partially arguments of these investigators.

In another work, Leipe et al. [45] demonstrated that Th17 cells play an important role in inflammation in human autoimmune arthritides, both at the onset and in established disease. They claim that the levels of IL-17 are connected to the systemic disease activity at both the onset and the progression of RA.

Taken together, these data suggest that IL-17 may be a key activator of T cell-driven inflammation and thus may contribute to the pathogenesis of RA [46, 47].

It can be asserted that IL-17 represents a member of the proinflammatory cytokine family produced by RA synovium and inhibited by some Th2 cytokines. In this way, IL-17 contributes to the active, proinflammatory pattern characteristic to RA, whose production and function are regulated by IL-4 and IL-13.

Reduction of synovial inflammation may be protective through a direct effect on IL-17, inducing proinflammatory effects. Thus, IL-17 appears to represent a target for treatments of RA [48, 49].

Overexpression of IL-17 has been shown to be associated with a number of pathological conditions. Since IL-17 was found at high levels in the synovial fluid around the affected cartilage in patients with inflammatory arthritis, it is assumed that this determines the direct effect on articular cartilage. IL-17 functions as a direct and potent inducer of matrix breakdown and an inhibitor of matrix synthesis in articular cartilage explants. Such findings have important implications for the treatment of degenerative joint diseases from arthritis [50].

It was observed that individuals in whom RA later developed had significantly increased concentrations of several cytokines, closer to the onset of symptoms, and that there exists a relationship between Th1, Th2, Treg, and Th17-cytokines and the presence of anti-CCP antibodies. Sensitivity, though, was not increased by the combination of anti-CCP antibodies and these cytokines [18, 41, 51, 52].

Because we know the statistical limits of the present study, the relatively small number of patients, we propose in the future longitudinal studies with regular serum analysis to determine more precise roles of IL-17, IL-13, and autoantibodies in RA pathogenesis.

## 5. Conclusions

With IL-13 and IL-17 serum concentrations increasing proportionally with the disease activity of eRA, the highest concentrations were in patients with severe activity disease. Based on the results of this study, we can conclude that the presence of higher IL-13, IL-17, IgM-FR, and anti-CCP serum levels in patients, compared to those of controls, confirms that these markers, found with high specificity, might be involved in the pathogenesis of eRA. IL-13 and IL-17 might be of better usefulness in the prediction of eRA activity status than IgM-RF and anti-CCP. Investigation of the association between cytokine profile and autoantibodies status may lead to prognostic and treatment decisions in eRA patients. The basis for the new therapies in patients with RA is represented by inhibiting the action of proinflammatory cytokines by using specific cytokine inhibitors or anti-inflammatory cytokines. The combination of IL-17 and anti-CCP autoantibodies may have the potential as biomarkers in early RA, especially for their clinical utility.

## Figures and Tables

**Table 1:** Clinical characteristics of the study subjects.

Character	Controls (*n* = 28)	eRA patients (*n* = 30)	*p* value
Age (yrs) (mean ± stdev)	52.36 ± 13.38	55.77 ± 10.87	NS (*p* = 0.391)
Gender (male/female)	6/22	6/24	—
DAS28	—	4.80 ± 0.84	—
Mild (2.6 < DAS28 ≤ 3.2) (*n*)	—	5	
Moderate (3.2 < DAS28 ≤ 5.1) (*n*)	—	10	
Severe (5.1 < DAS28) (*n*)	—	15	
CRP (mg/L)	5.01 ± 2.22	16.97 ± 5.14	*p* < 0.0001
ESR (mm/h)	11.89 ± 6.24	33.60 ± 12.35	*p* < 0.0001

**Table 2:** CRP, ESR, and autoantibodies (IgG anti-CCP, IgG anti-cL, anti-RA33, and IgM-RF) concentrations in serum of patients with eRA and in the control group.

	Disease activity eRA					
	Severe		Moderate		Mild	
	Mean	95% CI	Mean	95% CI	Mean	95% CI
CRP (mg/L)	19.33	17.34–21.33	14.99	10.81–19.17	13.86	7.50–20.22
ESR (mm/h)	34.53	29.96–39.11	37.30	25.55–49.05	23.40	11.54–35.26

*Autoantibodies*						
IgG anti-CCP (U/L)	162.20	124.60–199.70	49.76	19.57–79.95	16.20	6.93–25.47
IgG anti-cL (U/L)	13.27	9.61–16.92	16.30	9.90–22.70	11.40	3.18–19.62
Anti-RA33 (U/mL)	15.80	11.15–20.45	19.40	10.91–27.89	12.20	9.96–26.37
IgM-RF (U/L)	99.27	72.28–126.30	33.50	11.33–55.67	26.80	6.27–59.87

**Table 3:** Correlations between IL-13 and IL-17 and eRA indices.

	DAS28	IL-13	IL-17	IgM-RF	Anti-cL	Anti-RA33	Anti-CCP	CRP	ESR
DAS28		*r* = 0.060	*r* = 0.168	*r* = 0.140	*r* = 0.341	*r* = 0.404	*r* = 0.075	*r* = 0.051	**r** = 0.967
		*p* = 0.753	*p* = 0.376	*p* = 0.459	*p* = 0.065	*p* = 0.027^*∗*^	*p* = 0.694	*p* = 0.788	**p** ≤ 0.0001^*∗*^

IL-13			*r* = −0.054	*r* = 0.206	*r* = −0.082	*r* = 0.071	*r* = −0.033	**r** = −0.334	*r* = 0.019
			*p* = 0.775	*p* = 0.274	*p* = 0.668	*p* = 0.711	*p* = 0.864	**p** = 0.041^*∗*^	*p* = 0.919

IL-17				*r* = 0.320	*r* = −0.049	*r* = −0.249	*r* = 0.231	**r** = 0.366	*r* = 0.197
				*p* = 0.085	*p* = 0.795	*p* = 0.184	*p* = 0.219	**p** = 0.047^*∗*^	*p* = 0.298

IgM-RF					*r* = −0.320	*r* = 0.162	*r* = 0.418	*r* = 0.294	*r* = 0.071
					*p* = 0.085	*p* = 0.391	*p* = 0.022^*∗*^	*p* = 0.115	*p* = 0.709

Anti-cL						*r* = −0.173	*r* = 0.052	*r* = 0.129	*r* = 0.274
						*p* = 0.362	*p* = 0.784	*p* = 0.496	*p* = 0.142

Anti-RA33							*r* = −0.140	*r* = −0.066	**r** = 0.385
							*p* = 0.460	*p* = 0.731	**p** = 0.035^*∗*^

Anti-CCP								**r** = 0.371	*r* = −0.005
								**p** = 0.044^*∗*^	*p* = 0.979

CRP									*r* = −0.020
									*p* = 0.916

*r*: Pearson correlation coefficient; ^*∗*^statistically significant correlations.

**Table 4:** Diagnostic performance of the investigated parameters.

Parameter	AUC accuracy	Cut-off value	*p* value	Sensitivity (%)	Specificity (%)	Youden index
IL-13	1.000	10.73	<0.0001	**100.00**	**100.00**	1.000
IL-17	0.902	9.40	<0.0001	**86.67**	**100.00**	0.866

*Autoantibodies*						
IgM-RF	0.981	9.50	<0.0001	**94.12**	**97.06**	0.910
Anti-CCP	0.947	12.50	<0.0001	**94.44**	**91.18**	0.856
Anti-cL	0.847	7.50	<0.0001	**83.33**	**76.47**	0.598
Anti-RA33	0.762	8.50	0.00062	**80.00**	**64.29**	0.422

*Markers of inflammation*						
CRP	0.969	8.70	<0.0001	**96.67**	**100.00**	0.966
ESR	0.948	20.50	<0.0001	**83.33**	**92.86**	0.762
